# Recent advances in diagnosing and managing nut allergies with focus on hazelnuts, walnuts, and cashew nuts

**DOI:** 10.1016/j.waojou.2022.100641

**Published:** 2022-04-11

**Authors:** Magnus P. Borres, Sakura Sato, Motohiro Ebisawa

**Affiliations:** a Department of Women's and Children's Health, Uppsala University, Uppsala, Sweden; b Department of Allergy, Clinical Research Center for Allergy and Rheumatology, National Hospital Organization Sagamihara National Hospital, Kanagawa, Japan

**Keywords:** Allergen components, Component resolved diagnosis, Food hypersensitivity, Molecular allergology, Nut allergies

## Abstract

Tree nuts are a powerful and common source of food allergens that induce IgE-mediated allergic reactions. Health authorities endorse the intake of tree nuts because they are regarded as nutritious. Allergic reactions to nuts can lead to severe and occasionally lethal reactions. Allergies to tree nuts are observed worldwide and are found in up to 4.9% of people in unspecific populations.

Over the last 2 decades, the rates of allergic reactions and anaphylaxis have increased in different countries. Most proteins implicated in tree nut allergic reactions are members of the lipid transfer protein, 2S albumin, vicilin, legumin, and oleosin protein families. Bet v 1 homologs and profilins are involved in pollen-related tree nut allergies. Systematic literature reviews and meta-analyses on the diagnostic accuracy of specific immunoglobulin E (sIgE) for commercially available nut components have recently been published. IgE testing of the storage proteins Cor a 14, Cor a 9, Jug r 1, and Ana o 3 increases diagnostic specificity in assessing hazelnut, walnut, and cashew allergies in children, respectively. The resolution of tree nut allergies has been reported; however, only a few studies are available in this regard. Complete avoidance of nuts is the safest approach for nut-allergic subjects. However, this is difficult to achieve and can result in a severely restricted diet. Patients can eat nuts that they know are safe at home, but should avoid them when eating out because of the risk of cross-contamination.

Nuts have become part of a modern healthy diet, and this enhanced consumption is reflected in an increased prevalence of nut allergies.

## General introduction to nut allergies

Tree nuts are common food allergen sources that induce IgE-mediated allergic reactions.^1^ An allergic reaction to nuts can cause severe and occasionally lethal results. Tree nut allergy is observed worldwide and is frequent in up to 0.05% to 4.9% of individuals, as reported in a systematic review.^2^

A tree nut is a fruit consisting of a hard nutshell protecting the kernel. The stringent botanic description is not fully consistent with what is normally acknowledged as a tree nut; the colloquial definition of "tree nut" is any edible kernel from a tree. "Tree nut" has, therefore, become a collective term used to describe nuts that grow on trees.^2^ Contrary to popular belief, peanuts are not tree nuts, but groundnuts, and, as such, they are classified as a legume.

Some tree nuts are closely phylogenetically associated, while some are more distantly related. This association is also mirrored by protein sequence similarity, which stipulates the molecular conditions for possible IgE cross-reactivity. The kernels usually contain a substantial amount of important nutrients needed to provide energy for growth, persistence, and propagation. For instance, tree nuts are exceedingly rich in seed storage proteins. Although nut intake is endorsed by health authorities because they are regarded as nutritious, it varies widely by region. The report of “Tree Nuts: World Markets and Trade” mentioned that nut import markets were more diverse than their local counterparts.^3^

A general increase in emergency department visits for anaphylaxis has been observed in the United States between 2005 and 2014.^4^ The greatest increase occurred with anaphylaxis due to tree nuts/seeds, where a 373.0% increase was observed during the study period. The Swedish study showed that reactions to cashews, specifically, increased over the studied 10-year period, whereas reactions to other nuts were stable over time.^5^ Sicherer et al reported that the number of children with self-reported tree nut or peanut allergies increased over 11 years, from 0.6% to 2.1%, while the prevalence among adults remained constant over the same time frame.^6^ The most recent report is from Canada, where anaphylaxis to tree nuts significantly increased over the period from 2011 to 2017.^7^ Interestingly, anaphylaxis to peanuts significantly decreased over the same period in this study. Several studies based on results of oral food challenge (OFC) indicated that a history of anaphylaxis was a risk factor for future anaphylaxis,^8^ and a high level of antigen-specific Immunoglobulin E (sIgE) is positively related to anaphylaxis.^9^

Most proteins involved in tree nut allergy belong to protein families of 2S albumins, 7S globulins (legumins), 11S globulins (vicilins), non-specific lipid transfer protein (nsLTP), pathogenesis-related (PR)-10, profilins and oleosins (Table 1, Table 2). These protein families have different biological functions.^10^ Component-resolved diagnostics (CRD) offers the potential to improve the diagnostic accuracy of specific tree nut allergies through measuring s-IgE to the proteins as a complement to measuring s-IgE to whole allergens.^11^^,^^12^

**Table 1:** Overview of family and biological function of allergenic proteins in hazelnut, walnut, pecan, cashew, and pistachio.

Superfamily	Family	Biological function	Hazelnut	Walnut		Pecan	Cashew	Pistachio
Prolamin	nsLTP	High stability to thermal and enzymatic treatment, but its stability is pH-dependent	Cor a 8	Jug r 3				
				Jug r 8				
	2S albumin	High stability to thermal and enzymatic treatment	Cor a 14	Jug r 1	Jug n 1	Car i 1	Ana o 3	Pis v 1
Cupins	Vicilins	Intermediate stability to thermal and enzymatic treatment	Cor a 11	Jug r 2	Jug n 2	Car i 2	Ana o 1	Pis v 3
				Jug r 6				
	Legumins		Cor a 9	Jug r 4	Jug n 4	Car i 4	Ana o 2	Pis v 2
								Pis v 5
Bet v 1-like	Bet v 1	Low stability to thermal, ultrahigh-pressure, and enzymatic treatment	Cor a 1	Jug r 5				
Profilin-like	Profilin	Intermediate stability to thermal and enzymatic treatment	Cor a 2	Jug r 7				
	Oleosin	Structural proteins of oil bodies	Cor a 12					
			Cor a 13					
			Cor a 15					

This table is made based on data from World Health Organization and International Union of Immunological Societies Allergen Nomenclature Sub-Committee (February 3, 2022), nsLTP, non-specific lipid transfer protein

**Table 2:** Allergens in almond, Brazil nut, coconut and macadamia.

Superfamily	Family	Almond	Brazil nut	Coconut	Macadamia
Prolamin	nsLTP	Pru du 3			
	2S albumin		Ber e 1		
Cupins	Vicilins			Coc n 1	Mac i 1
	Legumins	Pru du 6	Ber e 2		Mac i 2
Bet v 1-like	Bet v 1	Pru du 1			
Profilin-like	Profilin	Pru du 4			
	Oleosin				
Other		Pru du 5, Pru du 8, Pru du 10			

This table is made based on data from World Health Organization and International Union of Immunological Societies Allergen Nomenclature Sub-Committee (February 3, 2022), nsLTP, non-specific lipid transfer protein

Allergies to other nuts such as almonds (*Prunus dulcis*) and Brazil nuts (*Bertholletia excelsa*) will not be covered in this review even though these allergies are clinically important and several allergens in these nuts are found (Table 2). The diagnostic value of almond allergens, for example, is mainly unknown, and almond sensitization is difficult to interpret. The prevalence of almond allergy among people with tree nut allergy is estimated to be almost 50%, yet the prevalence of food-challenge-defined almond allergy is ≤2%.^13^ Recently, Kabasser et al showed that Pru du 6 (legumin) effectively discriminated almond-allergic patients from tolerant patients. Hopefully soon, allergen components for almonds and more nuts will be available for allergy testing.

This review describes the recent trends in the prevalence of nut allergies and highlights the recent advances in molecular allergy diagnosis using allergen components in the clinic with a focus on clinical utility and interpretation.

## Hazelnut allergy (Table 1)

Hazelnuts (*Corylus avellana*) belong to the family Betulaceae.^10^ In 2017, the world's production of hazelnuts (in shells) was one million tons. Hazelnuts are used in confections to prepare pralines, chocolate truffles, and hazelnut paste products.

There is a wide range of clinical symptoms that arise in the allergic response to hazelnuts. The mildest form is oral itching, and the most severe is anaphylaxis. Hazelnut allergy is frequent in individuals presenting with pollen-food allergy syndrome, a respiratory disorder associated with allergies to pollens, such as birch, hazel, or alder.^14^

*Prevalence of hazelnut allergy*

There seems to be an important topographical and age-linked variation in the severity of hazelnut allergy symptoms. This variation can be seen between Europe and Japan, as well as within Europe itself. Allergy to hazelnut is stated to be the most prevalent tree nut allergy in Europe.^2^^,^^15^ According to a systematic review, hazelnut allergy is present in 17 % to 100% of people with tree nut allergies in Europe.^16^ The overall prevalence of doctor-diagnosed hazelnut allergy was found to be 1% in a European study conducted on school children enrolled from 8 European countries.^17^ The Pronuts study conducted in London, Geneva, and Valencia reported hazelnut allergy in 32% of nut-allergic individuals (n = 122).^18^ The inclusion criterion for this study was 1 or more nut or seed allergies, and hazelnut allergy was confirmed with OFC.

*Prevalence of sensitization to hazelnut allergen components*

The high prevalence of hazelnut allergy among people with allergies to other nuts and seeds is related to the high homology among the allergenic PR-10 proteins, which are responsible for the wide occurrence of cross-sensitization to multiple PR-10 proteins from different fruits, seeds, pollens, and nuts. In the Northern Hemisphere, most reactions to hazelnuts seem to be related to birch pollinosis, whereas non-pollen-related allergens play a significant role in hazelnut allergy in the Southern Hemisphere, signifying the presence of diverse forms of sensitization.^19^^,^^20^ As part of the PR-10 protein family, Cor a 1 is characterized as both inhalant and food allergen. In most cases, reactions related to this group of proteins are mild and are associated with oral allergy syndrome (OAS). This finding was reported in a recent study carried out in a birch-endemic area, where 97% of the study participants with OAS were sensitized to Cor a 1.04 and Cor a 1.0101 as a result of cross-reactivity with Bet v 1.^21^ The authors also stated that approximately 24% of young children and 50% of school-age children and adults with serious systemic reactions were sensitized to Cor a 1.04 or Cor a 1.0101. In 2002, Beyer et al recognized a protein in hazelnuts that seemed to belong to the legumin family. In 12 out of 14 (86%) patients with serious allergic reactions, this protein was recognized by serum IgE, which was named Cor a 9.^22^ The 2S albumin Cor a 14 and Cor a 9 were identified as indicators of serious events.

Among individuals with hazelnut allergies, 42% had sIgE to rCor a 2,^23^ while, in Southern Europe, hazelnut allergy is mostly linked to nsLTP, Cor a 8, and is related to serious responses.^24^ Although structurally similar to Pru p 3, the nsLTP from peach, there are variances in the epitope binding parts between the 2 molecules, which might cause partial cross-reactivity between Pru p 3 and Cor a 8.

*Clinical utility of hazelnut allergen components*

Hazelnut allergy has shown age-correlated sensitization profiles with different clinical consequences.^12^^,^^25^, ^26^, ^27^, ^28^, ^29^ Several studies have demonstrated that preschool children with hazelnut reactivity are often sensitized to Cor a 9 and/or Cor a 14.^12^^,^^25^, ^26^, ^27^, ^28^, ^29^, ^30^ Sensitization to 1 or both storage proteins has been related to immediate-type systemic responses in hazelnut allergic patients.^12^^,^^26^, ^27^, ^28^^,^^30^, ^31^, ^32^, ^33^, ^34^, ^35^, ^36^ Many European research groups have documented that sIgE testing for Cor a 14 in children resulted in a higher positive predictive value for hazelnut allergy than skin prick testing or sIgE testing to hazelnut extract, other hazelnut components, or Cor a 9.^21^^,^^30^^,^^32^^,^^34^^,^^36^^,^^37^ These results differ from those of a US study^28^ that demonstrated Cor a 9 to be similar to Cor a 14 in terms of positive predictive value as well as a Dutch study^27^ that reported Cor a 9 to be better than Cor a 14 for differentiating between patients with serious hazelnut allergy, mild hazelnut allergy, and no hazelnut allergy. In addition, Inoue et al demonstrated that IgE to Cor a 9 seemed to be a better marker of clinical reactivity to hazelnut in Japan than IgE to Cor a 14.^38^ Of the studies that found Cor a 14 to be a better marker than Cor a 9,^16^ a small subgroup of individuals with hazelnut allergy were negative for Cor a 14 and positive for Cor a 9, indicating the exceptional significance of each of these components in hazelnut allergy.^30^^,^^34^ The different results of these studies regarding the efficacy of Cor a 14 compared to Cor a 9 in identifying hazelnut reactive patients may have been due to the differences in study setup, demographics, and prevalence of other nut sensitizations.

Valcour et al studied hazelnut component sensitization patterns using a large sample size (n = 10,503) containing people with hazelnut extract-sIgE levels of 0.35 Â kU_A_/L or higher.^39^ In total, 89.6% of hazelnut-positive preschoolers were sensitized to Cor a 9, and 23.1% were sensitized to Cor a 14. Of this subgroup, 62% were sensitized to Cor a 9, but not to any of the other hazelnut components examined. Only 1.6% of these individuals were Cor a 14 monosensitized. Cor a 1 sIgE sensitization was much higher in adults than in children, especially in the northeastern United States. Cor a 8 sensitization was relatively constant (near 10%) across all ages.

In their systematic literature review and meta-analysis, Nilsson et al studied the diagnostic accuracy of sIgE at detecting hazelnut components to evaluate their contributions in diagnosing hazelnut allergy.^16^ Seven databases were examined for diagnostic studies on individuals suspected of having hazelnut allergy and when OFC had been performed. Seven cross-sectional studies and 1 case-control study were found, with 7 demonstrating data on children (n = 635) and 1with varied age groups.^12^^,^^27^^,^^28^^,^^30^^,^^32^^,^^36^^,^^40^ In children, the specificity of Cor a 14-sIgE at the 0.35 kUA/L cutoff was 81.7% (95% confidence interval [CI] 77.1, 85.6), and 67.3% (60.3, 73.6) for Cor a 9-sIgE. The specificities of Cor a 1-sIgE and hazelnut-sIgE were 22.5% (7.4, 51.2) and 10.8% (3.4, 29.8), respectively. The sensitivity of Cor a 1-sIgE (60.2% [46.9, 72.2]) was lower than that of hazelnut extract-sIgE (95.7% [88.7, 98.5]), while their specificities did not vary considerably. The authors concluded that sIgE to Cor a 14 and Cor 9 hazelnut storage proteins increases diagnostic specificity in evaluating hazelnut allergy at a young age.^16^ Combining testing of hazelnut extract with that of hazelnut storage proteins can enhance the diagnostic value.

Sensitization to hazelnuts is common among young asthmatics^41^ and can be a primary effect or a result of cross-reactivity. Johnson et al investigated the relationships between IgE antibody responses to hazelnut components, airway and systemic inflammation markers, and lung function parameters, and reported food hypersensitivity in a study of 408 asthmatic children and young adults in Sweden. The inclusion criteria were physician-diagnosed asthma with daily inhaled corticosteroids and/or oral leukotriene receptor antagonists for at least three months prior to study entry. Most of them were sensitized to hazelnuts (54%) and birch pollen (56%). Subjects sensitized to any of the hazelnut (Cor a 9 or 14) storage proteins were significantly younger (17.6% vs. 21.2%) and had higher levels of the fraction of exhaled nitric oxide (FeNO) (23.2 vs. 16.7 ppb) and B-Eos (340 vs. 170 cells/ml) than those with only pollen-related cross-reactive sensitization. FeNO levels were associated with IgE to storage protein levels in younger age groups. The authors concluded that sensitization to hazelnut storage proteins was related to higher levels of inflammation markers and food hypersensitivity symptoms in patients with asthma.

## Walnut allergy (Table 1)

Walnut (*Juglans regia*), a popular nut, is a member of the Juglandaceae family and is cultured worldwide, mostly in temperate climate zones. The nuts from all 24 walnut species are edible, but no more than two are economically important, specifically *Juglans regia* (also labeled as common, Persian, English, California, or Carpathian walnut) and *Juglans nigra* (Eastern black walnut).^42^

*Prevalence of walnut allergy*

Three percent of adult European residents are estimated to be sensitized to walnuts, varying from 0.1% in Iceland to 6% and 8% in Switzerland and Spain, respectively.^15^ Among children and adolescents with anaphylaxis to tree nuts, walnut is a common elicitor, accounting for 16% of the cases in Europe.^43^ In Chile, a study showed that allergy to walnut was the most prevalent food allergy in school-age children.^44^ Walnuts are the tree nuts most commonly responsible for triggering allergies in the United States, accounting for 37% to 48% of all tree nut allergies.^2^ In an Israeli OFC study, walnut allergy was found to be the most common allergy in individuals who were verified to have tree nut allergy; it was also found to be present in 74.6% of patients with a suspected tree nut allergy.^45^

*Prevalence of sensitization of walnut allergen components*

Currently, 8 allergens in walnut (Jug r 1–8) have been formally acknowledged (www.allergen.org). Jug r 1 is a member of the 2S albumin protein family, and 75% of walnut allergic patients develop sIgE against this protein.^46^ Jug r 2, a vicilin,^47^ is present in 60% of the patients and was subsequently classified as a major allergen. In 2004, a walnut LTP was characterized and named Jug r 3,^48^ sera from 37 of 46 (80%) walnut allergic individuals were found to contain IgE toward Jug r 3, and this specific binding was shown to be inhibited by Pru p 3, the LTP from peach. In the same decade, Wallowitz et al identified an additional allergenic walnut protein that was part of the legumin family and named it as Jug r 4.^49^ Of the 23 patients examined, 15 (65%) individuals had sIgE against Jug r 4. Recently, a PR-10-related protein was identified in walnuts and named Jug r 5,^50^ and 16 sera samples from birch pollen allergic patients with associated walnut allergy were screened. Although only 44% had tested positive for walnut allergy, 94% reacted to Jug r 5. When testing for IgE antibodies, Jug r 5 is clinically useful because it compensates for the low sensitivity of walnut extract testing in patients with reactions to walnuts. A vicilin-like cupin has been described in walnuts and named Jug r 6. Profilin has also been described in walnut and was named Jug r 7. Non-specific LTP type 2 was also identified and named Jug r 8 (www.allergen.org).

*Clinical utility of walnut allergen components*

Differentiating walnut sensitization from no symptoms of walnut allergies can be challenging. Although the established 0.35 kUA/L detection limit may result in good sensitivity, it results in low specificity.^51^ Nevertheless, higher levels (eg, sIgE levels >15 kUA/L) increase specificity, but result in poor sensitivity for walnut allergy.^52^^,^^53^ The clinical usefulness of component testing in walnut-allergic individuals is influenced by the population tested, taking into consideration both geography and patient age. Children present with true primary food sensitization more frequently than adults, which is usually distinguished by IgE targeting storage proteins. Grown-ups frequently exhibit cross-reactive sensitization, either exclusively or in addition to sensitization to storage proteins. This cross-reactive sensitization reflects IgE development to inhalant allergens and other foodstuffs and might be overshadowed by Jug r 5 in birch-populated areas such as northern Europe, or Jug r 3 in southern Europe, where sensitization to peach allergen Pru p 3 is prevalent. Sensitization to Jug r 1 is frequent in walnut-allergic patients from the United States, the United Kingdom, and central/northern Europe, while sensitization to Jug r 3 is prevalent in Italian and Spanish inhabitants, and sensitization to Jug r 5 in common in Swiss inhabitants.^46^^,^^48^^,^^54^^,^^55^ Researchers from Japan and the United Kingdom have demonstrated that Jug r 1 is better than walnut IgE in differentiating walnut allergy from sensitization.^55^^,^^56^ Elizur et al found that increased IgE levels to walnut molecules were most frequently found for Jug r 1, in their cohort. Sensitization to Jug r 4 and vicilins was also common.^51^ A level of IgE to Jug r 1 > 0.35 kUA/L was better than all other IgE levels at identifying walnut allergy, establishing an area under the curve (AUC) similar to that of walnut IgE with lower sensitivity but higher specificity. Joint practice of an IgE level of >0.35 kUA/L to Jug r 1 or 4 offered the ultimate accuracy for identifying walnut allergy with a sensitivity of 0.98, although maintaining a specificity of 0.73.

**Fig. 1:**
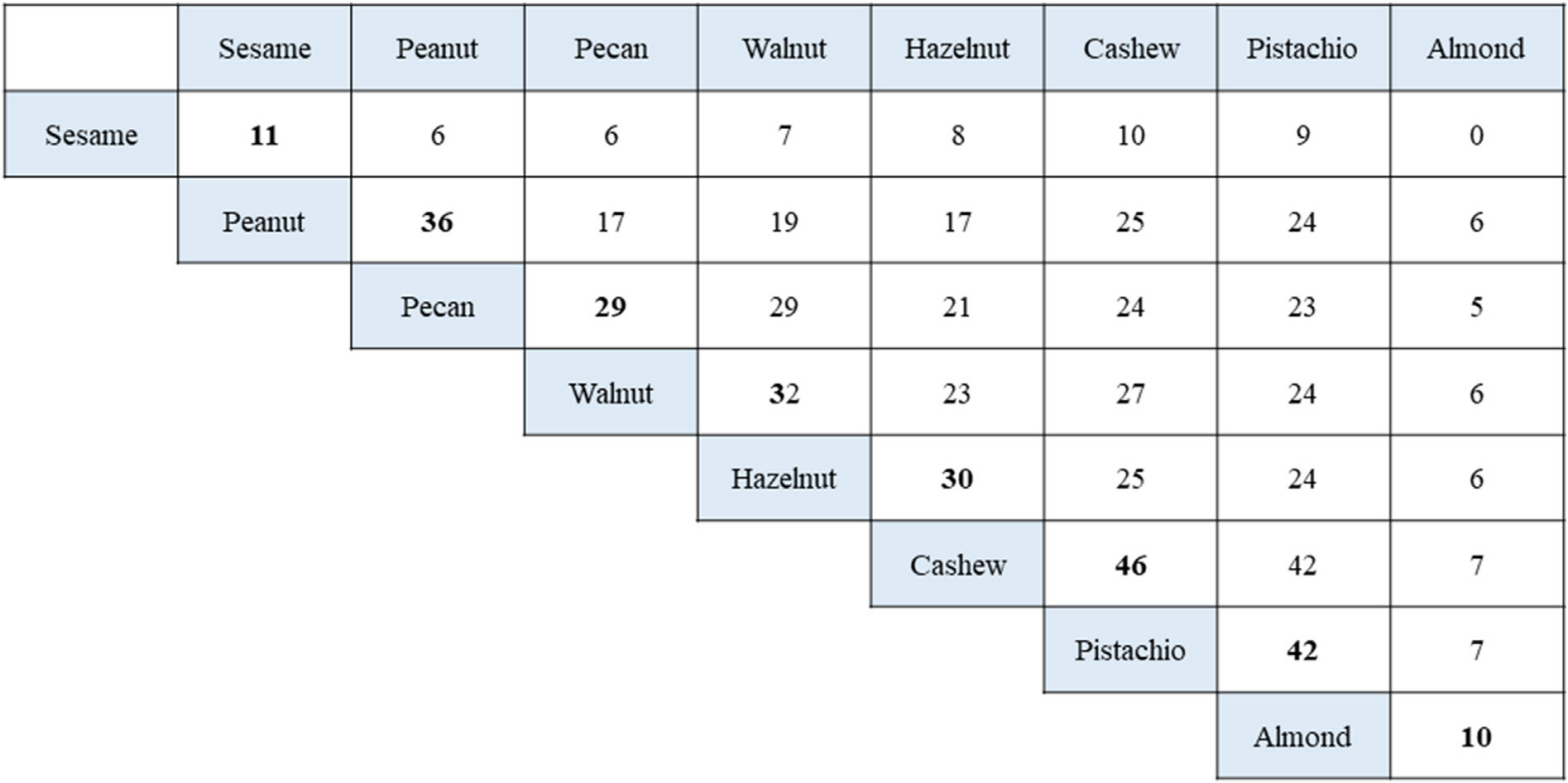
Concurrent occurrences of food allergies. Sixty patients with multifood allergies were placed in a double-blinded, placebo-controlled food challenge with different food items based on their clinical history and sensitization patterns. The graph shows the number of patients who reacted to sesame, peanut, pecan, walnut, hazelnut, cashew, pistachio, or almond. The number of subjects who were allergic to a particular food (diagonal) and any pairwise combination of 2 food allergens (intersection of column and row foods) are shown. Fig. 1 was modified from that of Andorf et al.^58^ This was printed with written permission from the authors

CRD has been previously demonstrated to recognize individuals who are at risk of more serious reactions.^57^ Andorf et al illustrated that sensitization to 2S albumin was related to increased digestive reactions (Fig. 1).^58^ Swiss researchers found that elevated concentrations of IgE antibodies to each of the walnut storage proteins were related to more serious events when performing walnut challenges.^54^ Elizur et al. found that high IgE antibody concentrations to Jug r 1 indicated the prognosis of more serious events, demanding injectable epinephrine.^51^

*Dual allergy to walnut and pecan*

**Fig. 2:**
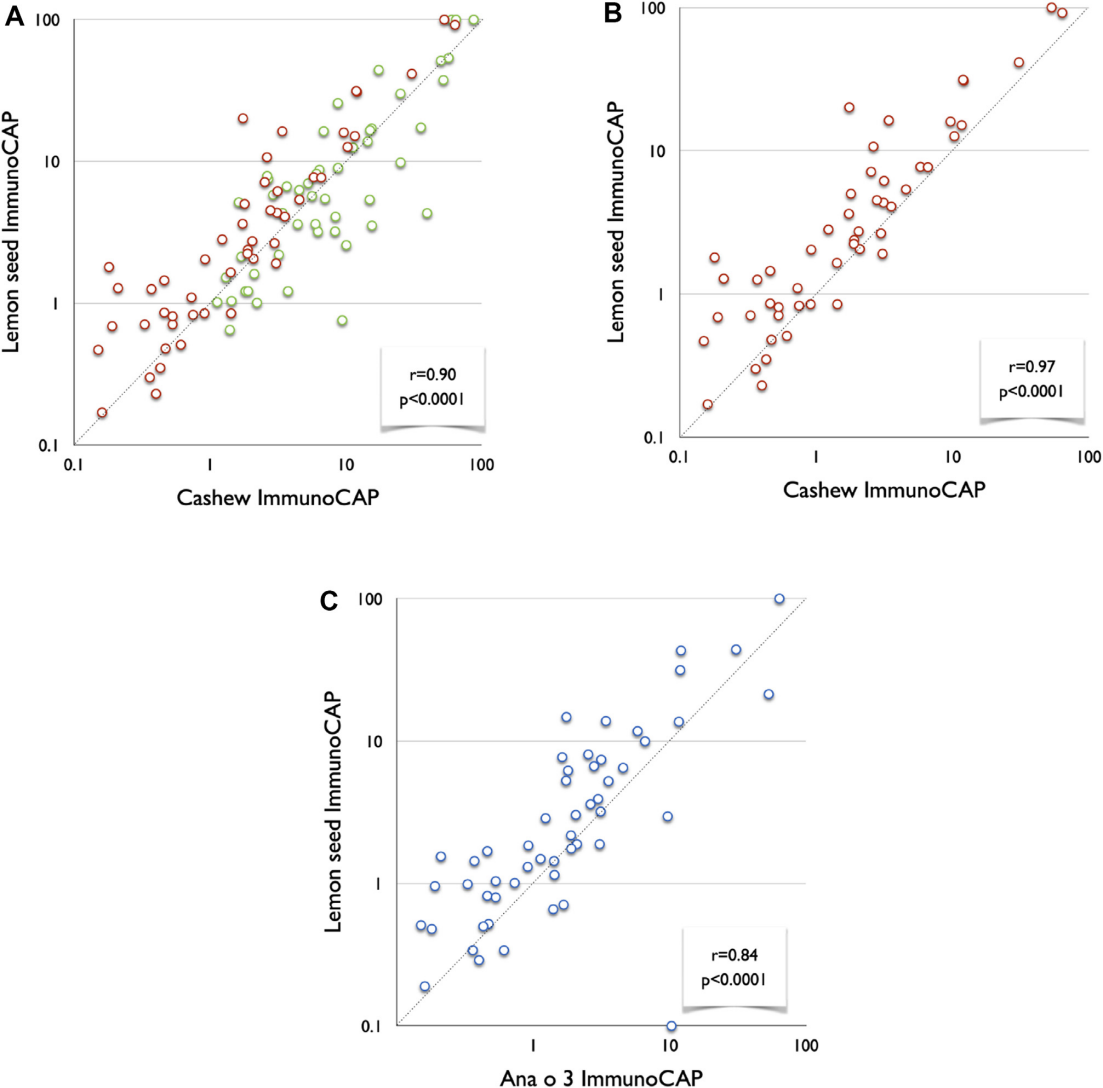
A-C; Specific-IgE levels in lemon seed, cashew nut, and Ana o 3. (A) S-IgE levels in lemon seeds and cashew nuts in the whole population. Green circles represent data from allergic children sensitized to pan-allergens (n = 52). Red circles indicate data from allergic subjects exclusively sensitized to seed-storage allergens (n = 51). (B) SIgE levels in lemon seed and cashew nut in allergic subjects solely sensitized to seed-storage allergens (n = 51). (C) SIgE levels for lemon seed and Ana o 3 in allergic subjects solely sensitized to seed-storage allergens (n = 51). These data were presented as a poster at The European Academy of Allergy and Clinical Immunology Annual Congress 2014,^81^ but have not been published in a journal. These data were printed with written permission from Savvatianos et al.

Pecan trees (*Carya illinoinensis*) are members of the Juglandaceae family in addition to walnuts. High rates of co-sensitization have been reported among walnuts and pecans (0.96).^58^ The great sequence identity among the 3 pecan allergens, namely, Car i 1 (2S albumin), Car i 2 (vicilin), and Car i 3 (legumin), with their corresponding proteins from walnut clarifies the commonly detected relationship between walnut and pecan allergies.^10^ Although OFCs to the co-allergenic nuts are not often carried out, there are few studies in this area. Andorf et al. found that all subjects reacting to pecans (n = 29) also reacted to walnuts. Only 3 of 32 subjects diagnosed with walnut allergies (9%) tolerated pecan (Fig. 2). These findings suggest that certain components are shared by these pairs of tree nuts, whereas others are exclusive to walnuts. Elizur et al confirmed this observation, showing that all subjects diagnosed with pecan allergy also reacted to walnuts. Two-thirds of the subjects reacting to walnut had an allergy to pecan. Subjects with dual allergies described more gastrointestinal reactions in the OFC than patients who were allergic to walnut only. Walnut allergy seemed more serious in subjects with dual allergies, as indicated by the considerably lower amount of walnut during the OFC procedure. The group also completed in vitro inhibition studies with walnut and pecan preparations to competitively bind walnut or pecan sIgE in the sera of walnut-only and dual walnut- and pecan-allergic individuals. The pecan extract was incapable of completely inhibiting IgE binding to walnut in the majority of walnut allergy cases but was capable of inhibiting IgE in pecan-tolerant subjects, signifying the existence of exclusive allergenic walnut epitopes. In subjects with dual allergies, serum preincubation with walnut extract completely inhibited IgE from attaching to itself and to pecan. These trials confirmed that all pecan-allergic subjects also reacted to walnut.

Diagnostically, IgE to Jug r 1 is the superior forecaster of walnut allergy; however, it offers no additional value in classifying patients with co-allergy to pecan. Instead, increased levels of IgE to Jug r 4 and low -and high-molecular-weight vicilins provided valuable evidence for differentiating solitary walnut from dual walnut pecan allergy. They concluded that subjects with an IgE antibody concentration of >0.35 kUA/L to Jug r 1 or Jug r 4 should be regarded as being allergic to walnut. Subjects with an IgE antibody concentration of >0.35 kUA/L to Jug r 4 would be interpreted as pecan allergies. It is yet to be proven whether walnut-only allergic patients develop dual allergies over time.

*New potential walnut allergy*

The allergenicity of the common walnut Juglans regia is well-documented, but little is known about the allergenicity of black walnut (*Juglans nigra*). Black walnut tree syrup is a developing gastronome foodstuff because of the reduction in maple syrup production owing to climate change. Presently, black walnut tree syrup is the main substitute tree syrup because of the extensive spread of the tree source and the high sugar content of sap. The production of black walnut tree syrup is expected to increase in the coming years. The findings of a pilot study by Lierl et al with 10 subjects showed that allergenic walnut proteins were not found in walnut tree syrup and proposed that black walnut tree syrup does not cause allergy in patients with a record of walnut allergy.^59^

## Cashew allergy (Table 1)

The cashew plant (*Anacardium occidentale*) belongs to the Anacardiaceae family and covers 9 species of the genus *Anacardium*.^60^ A. *Occidentale* is a tropical perennial tree that is recognized for the consumption value of its fruits and seeds. Cashew fruit (cashew apple) is an enlarged peduncle (pseudofruit) with a sugary flavor and scent. The cashew nut (seed) is cultivated on a hard shell at the bottom of the peduncle. Cashew nuts are used as ingredients in different processed food products, such as pesto, pastries, sweets, and confectionaries. Approximately 60% of cashew products are consumed as snacks after being roasted and salted.

*Prevalence of cashew allergy*

Similar to other nuts, the frequency of cashew nut allergy differs from country to country, with a peak incidence in the United States.^2^ It was estimated that cashew nut allergies had the second-highest incidence rate among US nut allergic subjects.^61^ Fleischer et al demonstrated a high prevalence of cashew nut allergies, with 30% prevalence in a double-blind placebo-controlled food challenge (DBPCFC) study in subjects with a prior diagnosis of tree nut allergies.^62^ Le et al reported a prevalence rate of 20% from the Netherlands in a similar study involving tree nut allergic adults.^63^ In a European multicenter study on anaphylaxis including children and adults, cashew was the sixth most common single food allergen,^64^ with large local variations in different regions.^43^ More than two-thirds of the patients in an Australian study with confirmed cashew nut allergy had suffered serious and dangerous effects, such as anaphylaxis.^65^ Interestingly, more patients in their study presented with a peanut allergy than with a cashew allergy, but anaphylaxis was more common in the cashew group (74.1% vs. 30.5%). Cashew allergens can provoke serious reactions in small amounts.^66^^,^^67^ In summary, cashew allergies are related to a high probability of anaphylaxis, as confirmed in multiple studies.

*Prevalence of sensitization to cashew allergen components*

To date, 3 cashew allergens have been formally acknowledged: Ana o 1, a vicilin protein; Ana o 2, a legumin protein; and Ana o 3, a 2S albumin (www.allergen.com).^68^, ^69^, ^70^ Van der Valk et al examined the IgE response to purified Ana o 1, 2, and 3 in the context of clinical outcomes.^71^ They found that all 3 proteins were independently prognostic of the result of food challenge tests in cashew-allergic subjects. Because of the extensive association between IgE levels and these components, the use of 1 of the components was recommended to be adequate.

*Clinical utility of cashew allergen components*

Ana o 3 has been documented as a very precise diagnostic indicator for cashew nut allergy, demonstrating higher specificity (94.4%) in contrast to the whole cashew (58.3%).^72^ Lange et al confirmed these findings, reporting that Ana o 3 was a better discriminator between allergic and tolerant subjects than sIgE to cashew (AUC: 0.94 vs. 0.78).^73^ Sato et al also found that Ana o 3 is a significant cashew allergen in Japanese subjects when performing cashew OFC in patients with cashew allergy suspicion.^74^

Blazowski et al examined the causative allergen in hospitalized children due to systemic allergic reactions and anaphylaxis.^11^ Anaphylaxis triggered by Ana o 3 (adjusted odds ratio = 15.0; 95% CI: 3.27 to 73.47) had the worst clinical presentation, including cardiovascular and serious respiratory symptoms. Almost 82% of patients with serious Ana o 3 anaphylaxis were sensitized only to this component and had no concomitant food sensitization. The authors concluded that monosensitization to Ana o 3 is associated with a high risk of severe anaphylaxis irrespective of other parameters.

In their systematic review, Betting et al evaluated the diagnostic tests for each tree nut and found that tests for cashew presented the greatest accuracy, with Ana o 3, in particular, showing a high diagnostic value.^75^ Considering the positive predictive value of the IgE test for Ana o 3, it might be a valuable marker for cashew allergy with high clinical sensitivity and specificity and a replacement for a considerable number of superfluous oral challenges.

*Dual allergy to cashew and pistachio*

Being part of the same Anarcadiaceae group, the kernels of pistachio (*Pistacia vera*) and cashew plants have a comparable protein expression profile. The extensive cross-reactivity of IgE-binding proteins has been verified by various research groups.^45^^,^^58^^,^^76^^,^^77^ Pistachio allergens characterized to-date are members of the protein families 2S albumins (Pis v 1), legumins (Pis v 2 and Pis v 5), vicilins (Pis v 3), and iron/manganese superoxide dismutase (Pis v 4).

Savvatianos et al showed that this widespread cross-reactivity between the two Anacardiaceae nuts has an impact on the clinical management of cashew allergy. They demonstrated an AUC of 0.97 when using Ana o 3 to predict pistachio allergy. This finding was based on the OFC, and Andorf et al verified this association with DBPCFCs in a US-based study. Interestingly, all subjects with pistachio allergy reacted to cashew challenges, and 42 of 46 subjects with cashew allergy reacted when challenged with pistachio. Amat et al also performed DBPCFC to investigate the usefulness of the Ana o 3 evaluation before OFC in pistachio. The level of s-IgE to Ana o 3 was better than that of s-IgE to pistachio (AUC 0.753 vs. 0.625).^78^

Elizur et al showed similar associations in an Israeli population. All pistachio-allergic subjects were allergic to cashew, and two-thirds of the individuals who were allergic to cashew had allergies to pistachio. This allergic unidirectionality suggests that particular components are common in these pairs of tree nuts, while others are unique to cashews and that subjects allergic to these dual pairs felt more digestive reactions on the OFC, possibly due to the 2S albumin component, than subjects who were allergic to cashews. Whether single cashew-allergic patients acquire dual allergies when they grow older is yet to be confirmed.

In summary, the high degree of clinically relevant IgE cross-reactivity demonstrated between the Anacardiaceae family 2S albumins in cashews and pistachios has led to the recognition of “the pistachio-cashew nut allergic syndrome,” as these nut allergies are highly correlated and do not predict allergies to other tree nuts.

*The Anacardiaceae and Rutaceae families are closely related*

The Rutaceae family (eg, lemon, tangerine, orange) is botanically closely related to the Anacardiaceae family, and cases of cashew-allergic individuals reacting to lemon and orange seeds have been described.^79^^,^^80^ Savvatianos et al studied the serologic cross-reactivity between lemon seed and cashew.^81^ Lemon and orange seed-sIgE levels were found to be highly correlated with IgE levels to cashew and pistachio, with an R-value ranging from 0.85 to 0.90 (Table 3). After eliminating sera from subjects sensitized to pan-allergens (LTP, PR-10, profilin, and CCD), the observed correlations were exceedingly high, with an R-value of 0.97 between cashew- and lemon-seed-IgE levels (Fig. 2A–C). The proteins from lemon seeds were purified and biochemically categorized. The identified allergens were used for IgE analysis in four Swedish cashew-allergic children with objective allergic symptoms that were suspected to be triggered by citrus seeds. Brandstrom et al found that the cashew-allergic children had IgE against novel citrus-seed allergens.^40^ The individuals described in the report were all cashew-allergic and known to tolerate the fruit pulp. When ingesting foods that contain the seeds of these fruits, they develop anaphylaxis. These studies and case reports need to be confirmed by others, but point to the fact that the described cross-reactivity to seed proteins is of clinical importance. We suggest that patients and families with severe cashew or pistachio allergies should be informed about the risk of accidental exposure to lemon kernels and, if possible, should avoid exposure. Practically, they should avoid chewing kernels and drinking juices containing crushed kernels.

**Table 3:** Relationship between specific IgE levels for lemon seed, orange seed, cashew, pistachio, and Ana o 3.

		Cashew	Pistachio	Ana o 3
All children (n = 103)	Lemon seed	0.9	0.9	0.79
	Orange seed	0.85	0.85	0.75
Children exclusively sensitized to seed storage allergens (n = 51)	Lemon seed	**0.97**	**0.94**	0.84
	Orange seed	**0.94**	**0.91**	0.84

Sera from 103 children (63 allergic to cashew, 63 allergic to pistachio, 5 with a positive challenge to orange/lemon seeds, and 11 children with a history highly suggestive of orange/lemon seed allergy) were analyzed for sIgE against cashew, pistachio, orange, and lemon seed extracts, and Ana o 3 by ImmunoCAP. Lemon and orange seed-specific IgE levels were found to be highly correlated with IgE levels to cashew and pistachio, with the r ranging from 0.85 to 0.90. After exclusion of sera from children sensitized to pan-allergens (LTP, PR-10, profilin, and CCD/n = 51), the observed correlations were exceedingly high, with r correlation coefficients >0.9, as shown in bold. These data were presented as a poster at The European Academy of Allergy and Clinical Immunology Annual Congress 2014^81^, but have not been published in a journal. These data were printed with written permission from Savvatianos et al. CCD, cross-reactive carbohydrate determinants; nsLTP, non-specific lipid transfer protein; PR-10, pathogenesis-related protein type 10

*Utility of multiplex CRD test in nut allergy*

The chip-based multiplex assay provides interpolated results from an internal calibration curve into semi-quantitative estimates of IgE antibodies as classes or grades. Their analytical sensitivity is generally less than that of singleplex tests. The majority of studies regarding the clinical utility of nut allergen components reviewed in this study have used simplex testing. The optimal application for multiplex testing resides in epidemiological studies. Multiplex testing is useful in the clinic when the physician wants to examine the sensitization profile to understand co-sensitization and cross-reactivity in patients with nut allergy.

Patients with birch pollen allergies often present with oral allergic symptoms associated with tree nuts. Hazelnuts contain PR-10, oleosins, nsLTPs and profilins. Hazelnuts, almonds, and walnuts contain PR-10 proteins and profilins, which may account for their cross-reactivity to pollen. Multiplex assays contain more components than those available for singleplex assays, even with a limited amount of serum. Therefore, it provides not only a sensitization profile to storage proteins but also a sensitization profile to allergens due to cross-reactivity to pollen.

## Conclusions

Establishing the tree nuts which cause the most clinically relevant allergies is multifaceted and difficult. The prevalence of individual allergies varies regionally, and the sensitization pattern changes over time. Current diagnostic methods for whole allergens seldom discriminate between sensitized and clinically allergic patients, and diagnosis has been heavily reliant on expensive and time-consuming OFCs. This is even more complicated for tree nut allergy because of the high prevalence of co-sensitization, requiring the need for improved *in vitro* diagnostics to make a conclusive diagnosis and prescribe avoidance strategies. Several studies and recent systematic reviews have shown that IgE testing for cashew, walnut, and hazelnut components is better than IgE testing for whole allergens in predicting the outcome of an OFC. Nut allergies affect at least one in 50 children and one in 200 adults, and most patients do not outgrow their allergies. The number of adults affected is likely to increase because of the cohort effect and the increased consumption of nuts. Nuts have become a part of a modern healthy diet, but measures to prevent adverse effects of the increased prevalence of nut allergies are of critical importance.

## Abbreviations

AUC, area under the curve; CI, confidence interval; CRD, component resolved diagnostics; DBPCFC, double-blind placebo-controlled food challenge; FDEIA, food-dependent exercise-induced anaphylaxis; IgE, immunoglobulin E; IgE-ab, IgE antibody; MA, molecular allergology; nsLTP, non-specific lipid transfer protein; OAS, oral allergy syndrome; OFC, oral food challenge; OIT, oral immunotherapy; PFS, pollen-food allergy syndrome; PR, pathogenesis-related; SPT, skin prick test; sIgE, specific immunoglobulin E.

## Funding sources

Some of our research activities were (partially) supported by a research grant from the Practical Research Project for Allergic Disease and Immunology from the Japan Agency for Medical Research and Development [AMED, 17ek0410019h0003].

## Availability of data and materials

Not applicable.

## Authors contribution

Magnus P Borres and Sakura Sato wrote the manuscript. Motohiro Ebisawa reviewed the manuscript.

## Ethics statement

Not applicable.

## Consent for publication

The authors' consented to the publication of this review.

## Declaration of competing interest

MPB is a medical director at Thermo Fisher Scientific. SS received a lecture fee from Mylan. ME received a lecture fee from DBV Technologies Scientific and Mylan. The authors did not receive funding for this article.
